# An intranasal recombinant NDV-BRSV F_opt_ vaccine is safe and reduces lesion severity in a colostrum-deprived calf model of RSV infection

**DOI:** 10.1038/s41598-022-26938-w

**Published:** 2022-12-29

**Authors:** Randy E. Sacco, Ignacio Mena, Mitchell V. Palmer, Russell K. Durbin, Adolfo García-Sastre, Joan E. Durbin

**Affiliations:** 1 Ruminant Diseases and Immunology Research Unit, National Animal Disease Center/USDA/ARS, 1920 Dayton Ave., Ames, IA 50010 USA; 2 Departments of Microbiology and Medicine, Icahn School of Medicine at Mount Sinai, One Gustave Levy Place, Box 1124, New York, NY 10029 USA; 3 Icahn School of Medicine at Mount Sinai, Global Health and Emergent Pathogens Institute, One Gustave Levy Place, Box 1124, New York, NY 10029 USA; 4 Infectious Bacterial Diseases Research Unit, National Animal Disease Center, USDA/ARS, 1920 Dayton Ave., Ames, IA 50010 USA; 5 Department of Pathology, Rutgers-New Jersey Medical School, 185 S. Orange Ave., Newark, NJ 07103 USA; 6 Department of Medicine, Division of Infectious Diseases, Icahn School of Medicine at Mount Sinai, One Gustave Levy Place, Box 1124, New York, NY 10029 USA; 7 Icahn School of Medicine at Mount Sinai, The Tisch Cancer Institute, One Gustave Levy Place, Box 1124, New York, NY 10029 USA; 8 Department of Pathology, Molecular and Cell-Based Medicine, Icahn School of Medicine at Mount Sinai, One Gustave Levy Place, Box 1124, New York, NY 10029 USA

**Keywords:** Live attenuated vaccines, Viral infection

## Abstract

Human respiratory syncytial virus (HRSV) is a major cause of severe lower respiratory tract disease in infants and the elderly, yet no safe, effective vaccine is commercially available. Closely related bovine RSV (BRSV) causes respiratory disease in young calves, with many similar features to those seen in HRSV. We previously showed that a Newcastle disease virus (NDV)-vectored vaccine expressing the F glycoprotein of HRSV reduced viral loads in lungs of mice and cotton rats and protected from HRSV. However, clinical signs and pathogenesis of disease in laboratory animals following HRSV infection differs from that observed in human infants. Thus, we examined whether a similar vaccine would protect neonatal calves from BRSV infection. Codon-optimized rNDV vaccine (rNDV-BRSV F_opt_) was constructed and administered to colostrum-deprived calves. The rNDV-BRSV F_opt_ vaccine was well-tolerated and there was no evidence of vaccine-enhanced disease in the upper airways or lungs of these calves compared to the non-vaccinated calves. We found two intranasal doses reduces severity of gross and microscopic lesions and decreases viral load in the lungs. Furthermore, serum neutralizing antibodies were generated in vaccinated calves. Finally, reduced lung CXC chemokine levels were observed in vaccinated calves after BRSV challenge. In summary, we have shown that rNDV-BRSV F_opt_ vaccine is safe in colostrum-deprived calves, and is effective in reducing lung lesions, and decreasing viral load in upper respiratory tract and lungs after challenge.

## Introduction

Respiratory infections remain a major cause of morbidity and mortality in children worldwide. Human respiratory syncytial virus (HRSV) is a significant cause of bronchiolitis, a common lower respiratory tract infection (LRI). The most severe LRI cases due to HRSV occur in infants and children less than a year of age, especially among those born premature or with underlying conditions^1,2^. The yearly occurrence of HRSV-associated disease is estimated to exceed 33 million cases in children younger than 5 years of age with more than 100,000 deaths^3^. BRSV is a closely related virus which causes respiratory disease in young calves and is a naturally occurring infection with many parallels to RSV infection in humans, including similar age dependency, and gross and microscopic lesions^4,5^. Thus, BRSV infection in calves can serve as an excellent model of RSV infection in children and to further test new vaccines that have proven to be efficacious in laboratory animal models prior to their use in human clinical trials. To date, there is no effective, commercially available vaccine for HRSV, and BRSV vaccines while available, have limited efficacy.

Human and bovine RSV are single-stranded, negative sense RNA viruses with approximately 15.2 kb genomes. Viral RNA is transcribed into 10 subgenomic RNAs encoding 11 proteins, with M2 gene encoding M2-1 and M2-2. There are 3 transmembrane surface glycoproteins, fusion (F), attachment (G) and small hydrophobic (SH) and a non-glycosylated matrix (M) protein. While antibody responses in humans are made to several HRSV proteins, the predominant neutralizing epitopes are found in the F and G proteins, with F known to contain the most important neutralizing antibody epitopes^6^. Similar to humans, calves mount antibody responses to several BRSV proteins, with the primary targets being F, G, and NP proteins^7^. Passive immunization studies in laboratory rodents have shown that administration of neutralizing IgG provides protection from HRSV lung replication^8,9^. On the other hand, the IgA response to HRSV is often transient and neutralizing IgM and serum IgG are likely more important in long-term protection against RSV^10^. An important role for neutralizing antibodies against the F protein in the response to HRSV has been proven in studies showing the effectiveness of palivizumab, an F-specific monoclonal antibody, at reducing disease severity in infants with HRSV^11–13^. In fact, the compelling clinical evidence that RSV F-specific antibody can protect against disease has led research into development of more potent passive protective antibodies by defining new F epitope targets^6^.

A lack of robust induction of type I IFN responses and resultant downstream antiviral mediators has been shown to play a role in failure of the host response to RSV infection. These type I IFN responses are known to be inhibited by HRSV and BRSV, both of which have evolved specific strategies to inhibit the IFN-induced cellular responses that are dependent upon nonstructural (NS) proteins^14,15^. As is characteristic of pneumoviruses, these viruses have two genes that encode for NS proteins. It has been shown that NS1 and NS2 proteins cooperatively mediate resistance of BRSV and HRSV to IFN-stimulated responses in a species-specific manner^16,17^. Recently, a recombinant Newcastle disease virus (rNDV) containing the RSV fusion (F) protein was tested in mice and cotton rats as a potential vaccine^18,19^. NDV is an avian paramyxovirus known to induce high levels of type I IFN levels. It is nonpathogenic in humans and mice, but can infect mammalian cells resulting in an abortive infection due to the induction of a strong local antiviral innate response^20,21^. The novel rNDV-F vaccine was shown to be efficacious in both rodent models protecting them from RSV challenge. However, as clinical signs and pathogenesis of the disease in these laboratory animals following HRSV infection differs from that observed in human infants, it was of interest to test a similar vaccine construct in a neonatal calf BRSV infection model. In order to examine vaccine safety and optimize vaccine dosage, colostrum-deprived calves were selected for use in the present study. Colostrum-deprived calves are more likely to manifest vaccine-enhanced disease, compared to conventional calves. We found that two intranasal (i.n.) doses of rNDV-BRSV F_opt_ were safe and reduced severity of RSV clinical signs, gross and microscopic pathology, and largely eliminated RSV in the upper airway and lungs by day 7 postchallenge in this neonatal colostrum-deprived calf model of BRSV infection.

## Results

### Preliminary vaccine trials

**Figure 1:**
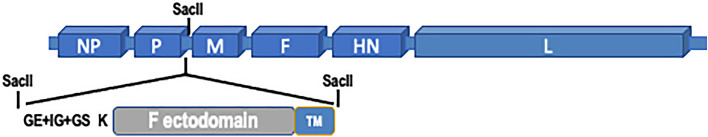
Construction of a recombinant NDV expressing a codon-optimized bovine RSV F protein. A functional transcription unit was inserted in the pNDV (LaSota strain) rescue plasmid at a unique restriction site (SacII) between the genes P and M. The additional transcription unit contains: NDV regulatory sequences Gene end-Intergenic-Gene Start (GE + IG + GS), Kozak sequence (K), a codon optimized synthetic sequence encoding the ectodomain of the bovine RSV F protein fused to the transmembrane and cytoplasmic domains (TM) of the NDV F protein.

In earlier preliminary studies (n = 3 independent experiments with 24 calves), a single intranasal dose of 1 × 10^8^ pfu rNDV-BRSV F_opt_ (Fig. 1) was tested as a vaccine. However, this single dose failed to provide protection from clinical signs of BRSV; moreover gross lung lesions at day 7 of BRSV challenge of vaccinated calves and calves receiving allantoic fluid were similar. Therefore, the decision was made to increase the dose to 5 × 10^8^ pfu rNDV-BRSV F_opt_ and to provide two immunizations. The increase in dose was calculated based on body weight of calves relative to the dose and body weight of laboratory rodents in which the rNDV-RSV F vaccine had been originally tested.

### Pre-challenge/vaccination

In screening serum samples of colostrum-deprived calves for BRSV antibodies, it was determined that one calf had received colostrum and this animal was not included in the analyses of neutralizing titers or immune mediators. One colostrum-deprived calf in the unvaccinated control group developed severe gastrointestinal and other health issues unrelated to BRSV within two weeks of arrival, and based on the recommendation of the clinical veterinarian overseeing these studies, this animal was euthanized. Overall, the vaccine was well-tolerated and there were no adverse reactions in any groups of calves following vaccination with allantoic fluid, rNDV empty vector, or rNDV-BRSV F_opt_.

### BRSV challenge and clinical signs

All calves were monitored daily and for clinical signs following BRSV challenge. Several animals in the BRSV allantoic group or BRSV wtNDF-empty vector groups exhibited elevated temperatures (scores 2–3, on a scale of 0–3) of at least 2 days duration, with one BSRV allantoic fluid and one BRSV + wtNDV-empty vector vaccinated calf having two episodes of 2 days duration. No calves in the BRSV rNDV-BRSV F_opt_ vaccinated cohort exhibited an elevated temperature score of 2 that lasted for more than 1 day. All together, 5/6 calves in the BRSV allantoic fluid group, 4/6 calves in the BRSV + wtNDV-empty vector vaccine group and 2/6 calves in the BRSV + rNDV-BRSV F_opt_ vaccinated group were observed by animal care staff to exhibit one or more clinical signs during these studies. No clinical signs of respiratory disease were observed in the uninfected calves during the challenge period. However, one calf in the uninfected control group exhibited a cough and elevated temperature of 2 days duration approximately one month before other cohorts in that experiment were challenged.

### Gross pathology is reduced in vaccinated calves

**Figure 2:**
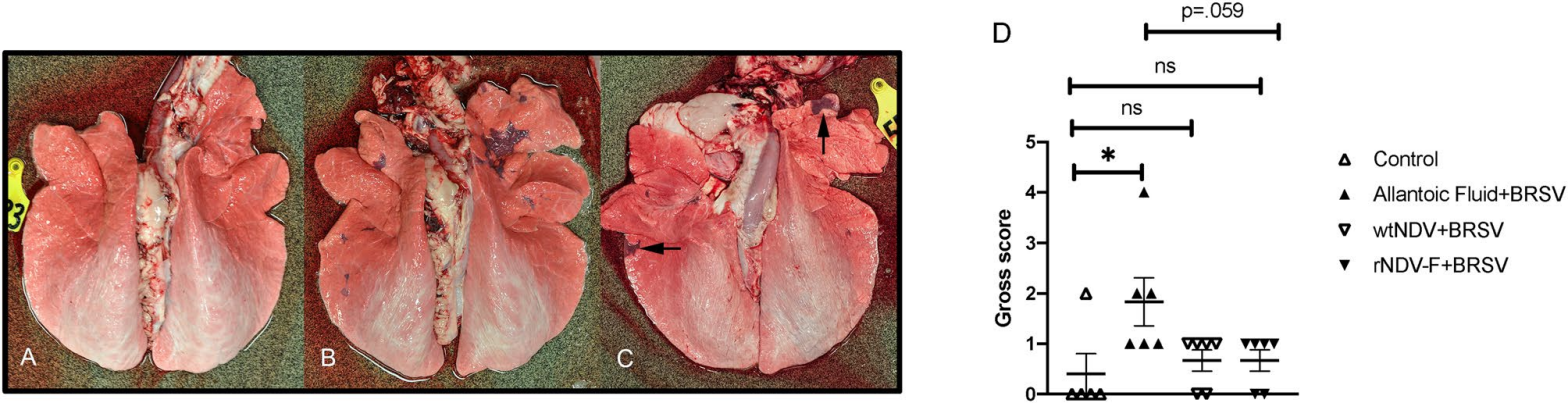
Reduced gross lesions in lungs of rNDV-BRSV F_opt_ vaccinated colostrum-deprived calves. Representative image of lung of non-vaccinated, non-infected calf (**A**). Gross lesions observed at day 7 postinfection with BRSV of a colostrum-deprived calf vaccinated with allantoic fluid (vaccine control) consisting of bilateral, multifocal to coalescing areas of firm, deep plum-red areas of consolidation and atelectasis, with lesions generally present in both cranial and caudal lung lobes (**B**). Lesions were similar, but much reduced in rNDV-BRSV F_opt_ vaccinated calves (**C**). While present (arrows), they are generally limited to cranioventral aspects of right and/or left cranial lobes. In (**D**), the overall gross pathology scores of each group of calves for each individual animal are shown. Statistical analyses were performed using ANOVA. *p < .05, **p < .01.

Representative lungs from control calves are shown in Fig. 2A. Gross lesions of colostrum-deprived BRSV/allantoic fluid calves (Fig. 2B) were similar to those we have previously described for conventionally-reared calves^4,22^ and consisted of unilateral or bilateral, multifocal to coalescing areas of firm, deep plum-red areas of consolidation and atelectasis ranging in size from 1 to 5 cm. Such lesions were generally present in both cranial and caudal lung lobes (Fig. 2B). Lesions were similar, but much reduced in rNDV-BRSV F_opt_ vaccinated calves (Fig. 2C). These lesions were present, but usually limited to cranioventral aspects of right and/or left cranial lobes. In Fig. 2D, the overall gross pathology scores of each group of calves for each individual animal are shown. A significant increase in gross lung lesion scores was seen in BRSV challenged-calves receiving allantoic fluid, whereas they were reduced in calves vaccinated with wtNDV or rNDV-BRSV F_opt._

### Reduced microscopic pathology in vaccinated calves

**Figure 3:**
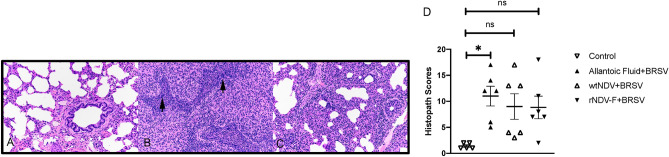
Reduced microscopic pathology in lungs of rNDV-BRSV F_opt_ vaccinated colostrum-deprived calves. Representative image of lung of non-vaccinated, non-infected calf (**A**). Significant microscopic lesions are shown in a representative photomicrograph from a non-vaccinated BRSV challenged calf (**B**). Alveolar septa are thickened due to congestion and infiltrates of macrophages, lymphocytes and neutrophils. Bronchioles are lined by attenuated epithelium with multifocal regions of epithelial cell loss. Bronchiolar lumens are variably filled with neutrophils, sloughed epithelial cells, debris and low numbers of multinucleated syncytial cells. Lesions in calves vaccinated with rNDV-BRSV F_opt_ (**C**) were primarily those involving alveolar septa (congestion, cellular infiltrates) with less involvement of bronchioles compared to non-vaccinated calves. In (**D**), histological scores are shown for each individual animal in each treatment group. Statistical analyses were performed using ANOVA. *p < .05, **p < .01.

Lungs from control calves had open alveoli, minimal insterstitial expansion and bronchioles with normal epithelium and clear lumens (Fig. 3A). Microscopic lesions in non-vaccinated BRSV challenged calves (Fig. 3B) were similar to those reported by us previously^4,22^. Alveolar septa were thickened due to congestion and infiltrates of macrophages, lymphocytes and neutrophils. Multiple bronchioles were lined by attenuated epithelium with multifocal regions of epithelial cell loss. Bronchiolar lumens were variably filled with neutrophils, sloughed epithelial cells, debris and low numbers of multinucleated syncytial cells. Lesions in calves vaccinated with rNDV-BRSV F_opt_ (Fig. 3C) were primarily those involving alveolar septa (congestion, cellular infiltrates) with less involvement of bronchioles compared to non-vaccinated control calves. In Fig. 3D, histological scores are shown for each individual animal in each treatment group. The histological scores of 4/6 calves in the allantoic fluid/BRSV challenge group were 12 or higher, whereas 4/6 BRSV + rNDV-BRSV F_opt_ vaccinated calves exhibited scores of less than 8. A significant increase in microscopic lung lesion scores was seen in BRSV challenged-calves receiving allantoic fluid, whereas they were reduced in calves vaccinated with wtNDV or rNDV-BRSV F_opt_.

### Reduced virus shedding in BRSV rNDV-BRSV F_opt_ vaccinated calves

**Figure 4:**
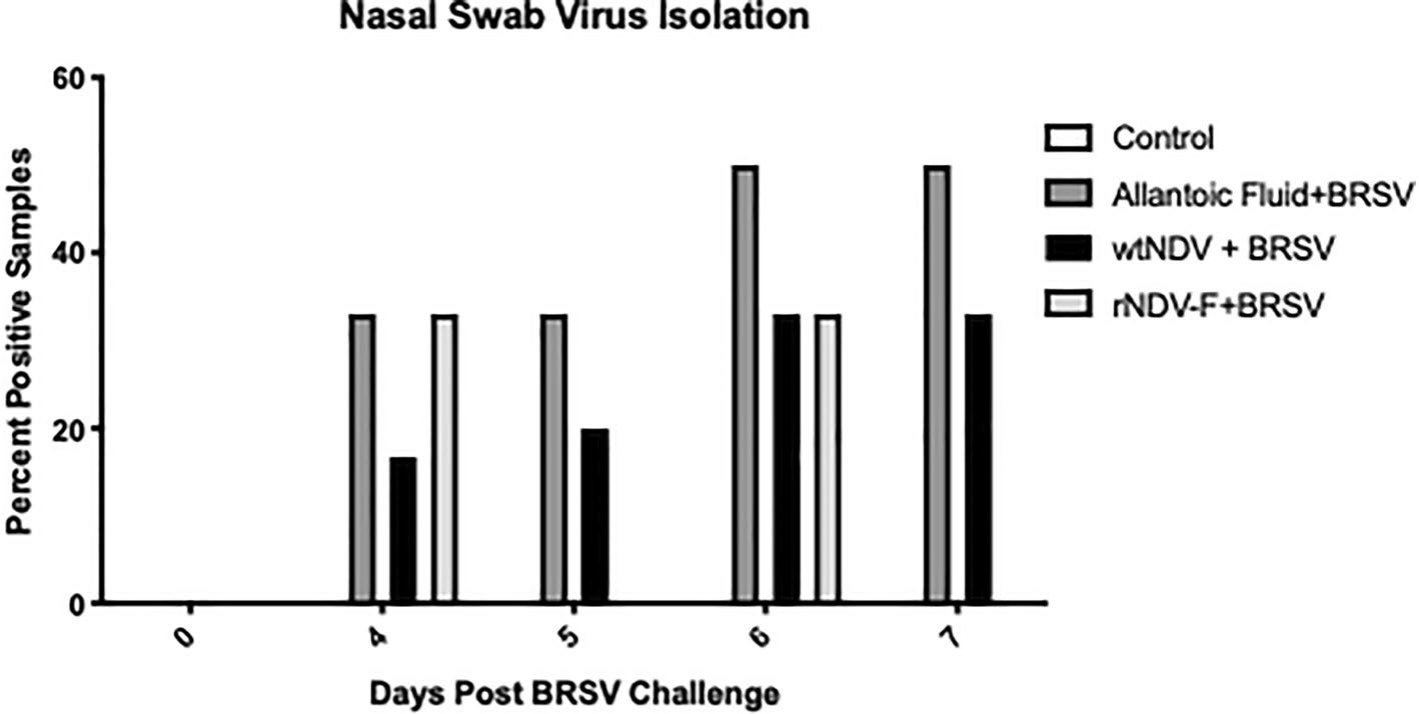
Reduced virus shedding of rNDV-BRSV F_opt_ vaccinated colostrum-deprived calves. Treatment groups of calves included: unvaccinated, uninfected controls (n = 5); intranasal (i.n.) allantoic fluid vaccinated (mock vaccine control) and BRSV challenged (n = 6); i.n. wtNDV-vaccinated and BRSV challenged (n = 6); i.n. rNDV-BRSV F_opt_ vaccinated and BRSV challenged (n = 6). Prior to vaccination, weekly following vaccination, and each day after experimental challenge, nasal swab samples were collected and placed in MEM containing antibiotics/antimycotics. Samples were vortexed and clarified by centrifugation and supernatants used to inoculate BT cells for virus isolation. Daily observations for cytopathic effect were conducted and recorded for 7 days following inoculation of cells. Results shown are the percentages of calves in each group which were positive for virus isolation.

Nasal swabs were collected prior to vaccination and weekly thereafter for virus isolation. We did not isolate virus from nasal swabs of any calves prior to BRSV challenge. The first BRSV isolation was observed on day 4 in all BRSV challenge groups (Fig. 4). On days 5 and 7 postchallenge, no BRSV was re-isolated from BRSV rNDV-BRSV F_opt_ vaccinated calves, whereas virus was isolated from calves receiving allantoic fluid or wtNDV empty vector. Over the 7 day challenge period, BRSV was isolated from 0/5 controls, 5/6 allantoic fluid vaccinated calves, 3/6 wtNDV vaccinated calves and 3/6 BRSV rNDV-BRSV F_opt_ vaccinated calves. Taken together, results of nasal swab virus isolation indicate reduced duration of BRSV shedding in BRSV rNDV-BRSV F_opt_ vaccinated calves compared to calves receiving an intranasal inoculation of allantoic fluid.

### Reduced viral burden in lungs of BRSV rNDV-BRSV F_opt_vaccinated calves

**Figure 5:**
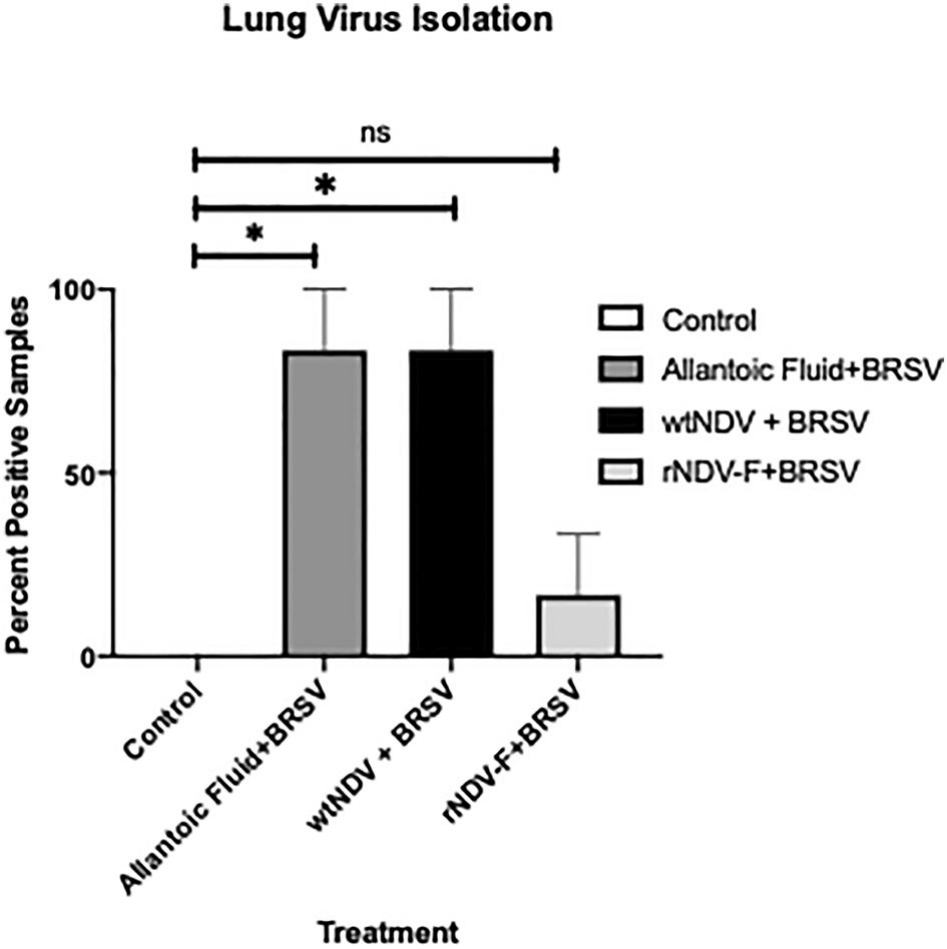
Reduced viral burden in the lungs of rNDV-BRSV F_opt_ vaccinated colostrum-deprived calves. Treatment groups of calves included: unvaccinated, uninfected controls (n = 5); intranasal (i.n.) allantoic fluid vaccinated (mock vaccine control) and BRSV challenged (n = 6); i.n. wtNDV-vaccinated and BRSV challenged (n = 6); i.n. rNDV-BRSV F_opt_ vaccinated and BRSV challenged (n = 6). Lung homogenates were prepared using tissue collected at necropsy and a 10% suspension used to inoculate BT cells for virus isolation. Daily observations for cytopathic effect were conducted and recorded for 7 days following inoculation of cells. Results shown are the percentages of calves in each group which were positive for virus isolation. Statistical analysis on percentage data was performed using Kruskal–Wallis ANOVA.

Lung tissue samples were collected from representative gross lesions at necropsy on day 7 for virus isolation. Homogenates of lung were prepared from tissue and a 10% solution used to inoculate BT cells. As seen in Fig. 5, virus was re-isolated from lungs of 5/6 calves receiving allantoic fluid and 5/5 calves receiving wtNDV empty vector as a vaccine, but only 1/6 calves vaccinated with BRSV rNDV-BRSV F_opt_. We did not isolate virus from the lung samples of any of the non-infected control calves. These data indicate a significant reduction in viral burden in the lung of calves receiving the intranasal BRSV rNDV-BRSV F_opt_ vaccine.

### Sustained increase in serum viral neutralization titers in BRSV rNDV-BRSV F_opt_ vaccinated calves

**Figure 6:**
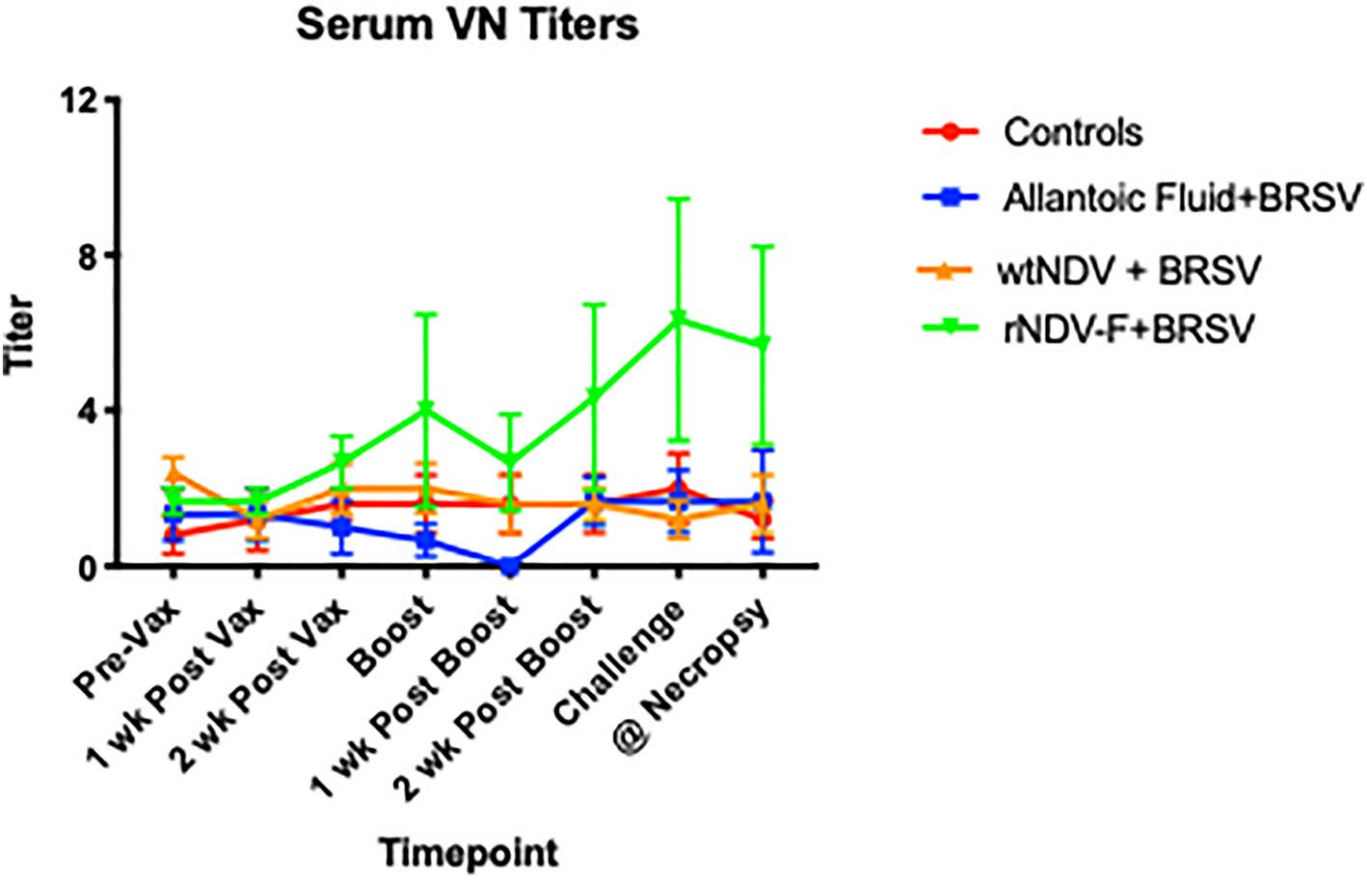
Serum neutralizing antibody titers measured following vaccination and BRSV challenge of colostrum-deprived calves. Sera was harvested from calves pre-vaccination and weekly following intranasal vaccination and diluted two-fold for determination of virus neutralization titers. Treatment groups of calves included: unvaccinated, uninfected controls (n = 5); intranasal (i.n.) allantoic fluid vaccinated (mock vaccine control) and BRSV challenged (n = 6); i.n. wtNDV-vaccinated and BRSV challenged (n = 5); i.n. rNDV-BRSV F_opt_ vaccinated and BRSV challenged (n = 6). Data are presented as mean titer per group. Note that the only group of calves whose mean titer was 1:4 or higher was the group receiving i.n. vaccination with rNDV-BRSV F_opt_ and this was sustained from boost to the end point of each of the 3 experiments.

Colostrum-deprived calves can mount antibody responses, but the peak titers of these calves are typically lower than observed for conventionally reared calves. Such was the case in the present study as shown in Fig. 6 compared to the response frequently observed in conventionally reared calves. It should be noted, however, that the only group of calves to exhibit a titer of 1:4 or higher at 3 wk post primary vaccination was the BRSV rNDV-BRSV F_opt_ vaccinated group. An increase in viral neutralization titers was seen at 2 and 3 wk after booster vaccination and sustained until necropsy day 7 post challenge with BRSV (4 wk after boost).

### Reduced CXC chemokine gene expression in lesioned lung samples from BRSV rNDV-BRSV F_opt_ vaccinated calves

**Figure 7:**
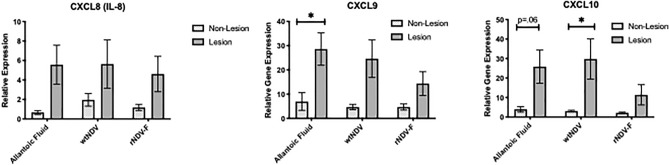
CXC chemokine gene expression in lung tissues of colostrum-deprived calves following BRSV experimental infection. Treatment groups of calves included: unvaccinated, uninfected controls (n = 5); intranasal (i.n.) allantoic fluid vaccinated (mock vaccine control) and BRSV challenged (n = 6); i.n. wtNDV-vaccinated and BRSV challenged (n = 5); i.n. rNDV-BRSV F_opt_ vaccinated and BRSV challenged (n = 6). Samples from lesioned and non-lesioned portions of lung were collected from calves 7 post-challenge with BRSV and placed in RNALater. The amount of each target gene was determined by quantitative real-time PCR with normalization to RPS9. Relative gene expression of each target was determined using the 2^-ΔΔCt^ method. ΔΔCt values ± SE were transformed (2^-ΔΔCt^) and data are presented as the target gene expression relative to control lung samples (n = 4). Statistical analysis was performed using ANOVA. *p < .05, **p < .01.

In infants with severe RSV bronchiolitis, it has been shown that CXC chemokines contribute to the observed pulmonary pathogenesis inducing leukocyte infiltration^23–25^. We had previously shown an increase in CXCL8 gene expression in the inflamed lung of the calf compared to that observed in non-lesioned lung similar to that observed in infants, but had not examined other CXC chemokines^4^. Thus, levels of CXC9 and CXCL10 chemokines were examined in BRSV rNDV-BRSV F_opt_ vaccinated calves. As shown in Fig. 7, there was increased mRNA expression of CXCL9 and CXCL10 in lesioned samples from BRSV challenged calves vaccinated with allantoic fluid or wtNDV compared to non-lesioned samples from those calves. In contrast, the gene expression of CXC chemokines in lesioned lung samples from BRSV-challenged rNDV-BRSV F_opt_ vaccinated calves was not significantly different from non-lesioned samples of those calves. Thus, the vaccine reduced the expression of inflammatory mediators typically associated with RSV bronchiolitis.

## Discussion

RSV infection continues to have a devastating impact in pediatric populations worldwide, as well as in immunocompromised and elderly patients. The primary target population in the development of an efficacious vaccine are infants less than six months old, but there is a further need to provide an effective vaccine for other at-risk populations. Testing vaccines in animal models has primarily occurred in mice and cotton rats that are semi-permissive for HRSV infection. We believe that there are several major advantages to the bovine model system for testing novel vaccines for RSV infection as we previously described^22,26^ that make it ideally suited as an additional animal model to test novel HRSV vaccines prior to accessing their safety and efficacy in human clinical trials. In the current study, we utilized colostrum-deprived calves to optimize the dose and number of immunizations for the rNDV-BRSV F_opt_ vaccine.

Of utmost importance in the development of new HRSV vaccines are pre-clinical vaccine safety data which ideally should be derived from multiple animal models prior to initiation of clinical trials in human patients. The importance of obtaining pre-clinical safety data has been at the forefront in the development of new HRSV vaccines since the disastrous historical results of clinical trials with a formalin-inactivated HRSV vaccine in the 1960s. This vaccine not only failed to induce protection against HRSV, but also induced an exaggerated clinical response in infants that received the vaccine^27–29^. Many of these vaccinated infants were hospitalized, with rates in one study approaching 80% compared to 5% of non-vaccinated infants^29^. Unfortunately, two infants died and HRSV was readily isolated from the lower respiratory tracts of these infants^29^. Therefore, it is imperative that new vaccines tested do not exacerbate clinical signs or lesion severity. In the present study, rNDV-BRSV F_opt_ vaccine was well-tolerated by calves in which no untoward clinical signs were noted following administration of primary or booster vaccines and there was no evidence of vaccine-enhanced disease in the upper airways or lungs of colostrum-deprived calves receiving the vaccine compared to the non-vaccinated control calves.

It is critical that upper airway infection is eliminated, as virus shedding during upper respiratory tract infection of healthy individuals is a key source of infection for at-risk individuals. In addition, otitis media in young children as a result of RSV infection in the upper airway is an important secondary consequence of infection and prevention of infection leading to this clinical condition would be a further important aim of vaccination. Moreover, RSV infections during the first two years of life may shape remodeling of the airways and contribute to the development of recurrent wheezing and asthma^30^. Certainly, therefore, elimination of lower respiratory tract infection to prevent severe disease manifestations remains a goal of RSV vaccine candidates. Therefore, any RSV candidate vaccine should eliminate both upper and lower respiratory tract infection. rNDV-BRSV F_opt_ vaccination eliminated viral shedding by day 7 postchallenge as measured by virus isolation from nasal swabs. Furthermore, BRSV was re-isolated from the lungs of only 1 of 6 calves receiving this vaccination. Thus, rNDV-BRSV F_opt_ vaccine virtually eliminated upper and lower respiratory tract infection in colostrum-deprived calves.

Neutralizing Abs targeting F and G are important targets in controlling RSV infection, with roles for both IgG and IgA isotypes. However, the importance of antibodies in controlling infection remains controversial, as experimental infection can be achieved even in the face of maternal antibodies. However, neutralizing activity in cord blood correlates with protection of infants from severe disease^31^. In addition, therapy with palivizumab, a neutralizing monoclonal Ab targeting F protein reduces protect high-risk infants from severe infections and reduces hospitalization rates. Further, motavizumab, a humanized IgG1 monoclonal antibody designed to have a higher affinity than palivizumab from which it was derived, significantly reduced RSV-associated disease in Native American infants^32^. Thus, an important primary immunological goal to be achieved with F-based vaccines is neutralizing activity^33^. In the present study, neutralizing antibodies were generated in serum of colostrum-deprived calves administered the rNDV-BRSV F_opt_ vaccine via the intranasal route.

There are studies which have shown that CXC chemokines can be detected in secretions during RSV bronchiolitis in infants^23,24^ and these mediators contribute to both inflammation and pathophysiology of disease^23–25^. Thus, a vaccine which can reduce the expression of these inflammatory mediators should decrease the severity of RSV bronchiolitis. We had previously shown in the calf model of RSV infection that CXCL-8 is upregulated in the lungs^4^, but had not examined other chemokines. In the present study, we observed that CXC chemokines were significantly higher in lesioned compared to non-lesioned lung samples in RSV-infected calves. Vaccinated calves had reduced CXC chemokine levels in lesioned lung samples compared to non-lesioned samples. Reduced expression of these chemokines likely played a role in decreased lesion severity and a reduction in cellular infiltration into RSV-inflamed lungs.

Some protective effects that we observed for vaccinated calves might be mediated at least in part by non-specific innate antiviral responses, as both the rNDV-BRSV F_opt_ vaccine and the wt-NDV empty control protected from gross and microscopic lesions, and reduced viral shedding. This is perhaps due to the high type I IFN inducing properties of NDV vectors^20,21^, which may provide non-specific innate protection. However, some other protective effects appear to be mediated by specific immunity and correlate with the induction of neutralizing antibodies, such as the reduction seen in viral load in the lungs and the induction of chemokines after challenge.

As there are currently multiple HRSV vaccines in phase 3 clinical trials, including mRNA and pre-F subunit vaccines, an assessment of advantages and disadvantages of the rNDV-BRSV F_opt_ candidate vaccine is warranted. The advantages of the rNDV vaccine platform and delivery route used in the present study are the following. Intranasal administration of modified live virus vaccines has been shown to induce mucosal immune responses and local delivery of antigen necessary to activate lung lymphocyte subpopulations and establish resident memory within the respiratory tract^34^, thereby protecting against the virus at its site of entry. Secondly, rNDV-vectored vaccines can be grown in embryonated eggs, using the same manufacturing methods and facilities as used to produce the seasonal influenza vaccine. Finally, NDV replication would exhibit adjuvant properties, whereas vaccines that require the use of an adjuvant are more costly and may exhibit secondary effects. The latter two advantages of this vaccine would result in reduced costs of production, which is critical, as HRSV vaccine manufacturing should allow for their optimal use in low and middle-income countries per WHO recommendations (https://www.who.int/publications/m/item/respiratory-syncytial-virus-vaccines-annex-2-trs-no-1024). One hypothetical disadvantage of the use of rNDV vaccines is the potential for recombination of RNA viruses. However, and in contrast to positive strand RNA viruses, negative strand RNA virus, such as NDV, have a negligible rate of recombination^35^. Natural infection of humans with NDV is not common, and NDV has a restricted replication in primates due to the induction of interferon responses. Therefore, the restricted replication reduces the opportunity for genetic exchange. Furthermore, NDV has a low level of sequence relatedness with circulating human non-segmented negative strand RNA viruses. There is no evidence that recombination between dissimilar NNSVs occurs. An additional possible disadvantage is the potential for instability of the inserted foreign gene. Previously, it was shown that inserts in non-segmented negative strand RNA viruses are very stable^36^. Therefore, the advantages of using rNDV-vectored vaccines clearly outweigh any potential disadvantages.

In summary, we have shown that two doses of rNDV-BRSV F_opt_ vaccine is safe in colostrum-deprived calves, reduces severity of lung lesions and decreases viral load in the upper respiratory tract and lungs. In future studies, it will be necessary to examine vaccination of conventionally reared calves in order to examine the effectiveness of vaccination in the presence of maternal antibodies and to measure neutralizing Ab and antigen-specific T cell responses in an animal with a robust, conventionally-developed immune system.

## Materials and methods

### rNDV-BRSV F_opt_ construct

A codon-optimized rNDV-BRSV F (rNDV-BRSV F_opt_) vaccine was constructed using methodology similar to that previously described for rNDV-HRSV F vaccine used in laboratory rodent models^18,19^. Briefly, the pNDV (LaSota strain) rescue plasmid was modified to insert an additional transcription unit containing a synthetic, codon-optimized ectodomain of the BRSV F protein fused to the transmembrane and cytoplasmic domains of the NDV F protein (Fig. 1). The plasmid was used to rescue infectious rNDV-BRSV F using a well stablished protocol. Presence of the insert in the viral RNA was confirmed by RT-PCR and expression of the BRSV F protein was confirmed by immunofluorescence (IF) and flow cytometry on infected Vero cells (not shown) and by IF on infected bovine turbinate (BT) cells (supp. Fig. S1). rNDV or rNDV-BRSV F_opt_ were amplified in embryonated chicken eggs for 10 days.

### Animals

For these studies, 24 neonatal colostrum-deprived calves were obtained from a local commercial dairy farm. The studies involved 3 replicated experiments with 8 calves per experiment. Upon arrival calves received a milk replacer diet according to manufacturer’s instructions (Purina Mills LLC, St Louis MO) twice daily and were transitioned to a calf starter diet. Ad libitum water was available. Calves were acclimated for approximately seven days prior to vaccination. In addition, prior to vaccination a serum sample was collected from each and sent to the Iowa State University Diagnostic Laboratory to screen for BRSV antibodies as confirmation that the calves had not received colostrum. All animal experiments were conducted in strict accordance with federal and institutional guidelines and approved by the USDA National Animal Disease Center Institutional Animal Care and Use Committee (ACUP# ARS-2017–615, approved 4/18/2019). This study is being reported in accordance with the ARRIVE guidelines.

### Vaccination

At arrival, calves were randomly assigned to one of 4 groups with calves in each group housed in separate rooms: unvaccinated, uninfected controls (n = 6); intranasal (i.n.) allantoic fluid vaccinated (mock vaccine control) and BRSV challenged (n = 6); i.n. rNDV-vaccinated and BRSV challenged (n = 6); i.n. rNDV-BRSV F_opt_ vaccinated and BRSV challenged (n = 6). Vaccinated calves either received 5 × 10^8^ pfu rNDV or rNDV-BRSV F_opt_, or a similar volume (2.5 mL per nostril) of allantoic fluid. Three weeks after the primary vaccination, calves were given a booster i.n. immunization.

### Nasal fluid samples

Nasal secretion samples were collected pre-vaccination and weekly following vaccination and on day of necropsy as previously described (McGill et al. 2018). Briefly, a sterile sponge pre-soaked in 1 ml of PBS was inserted into the nasal cavity for 5 min. The sponge was then placed into a sterile tube.

### Serum samples

Blood samples were collected weekly pre-vaccination and post-vaccination and on the day of necropsy.

### BRSV inoculum and challenge model

Low-passage virus used in preparation of the inoculum had been re-isolated from a lesioned-lung section of an infected calf. When BRSV-induced cytopathic effect on bovine turbinate cells reached 90% on fourth passage, flasks were frozen, and thawed twice. Media from flasks were pooled and cellular debris removed by centrifugation. Filtered supernatants were aliquoted and stored at −80 °C. A standard plaque assay was used to determine the tissue culture infective dose (TCID_50_/ ml) of a representative aliquot. In addition, the viral inoculum was determined to be BVDV free by PCR. Three weeks following booster vaccination, calves were challenged via aerosol with 5 ml of BRSV strain 375 inoculum containing 10^5^ TCID_50_/ml, as previously described^4,22^.

### Clinical signs

Following challenge, calves were examined twice daily for clinical signs. A scoring system from the University of Wisconsin School of Veterinary Medicine was used for evaluation of clinical signs in these studies (https://www.vetmed.wisc.edu/fapm/wp-content/uploads/2020/01/calf_respiratory_scoring_chart.pdf). Rectal temperatures were recorded and additional scores were given for cough, nasal discharge, eye discharge, and ear tilt, and recorded for each calf.

### Necropsy and pathological evaluation

On day 7 day following challenge, the calves were euthanized using sodium pentobarbital. The thorax was opened and the lungs evaluated for gross lesions using a scoring system similar to that previously described^22^ and shown in supplementary Table 1. Left cranial, right cranial, and right caudal lung lobes, tracheobronchial lymph node (TBLN), medial retropharyngeal LN, nasal turbinates, and trachea tissue samples were collected from each calf at necropsy for histopathological evaluation. Tissues samples were placed in 10% neutral buffered formalin for 24 h fixation and transferred to 70% ethanol until further processing. Routine paraffin-embedment processing techniques were used on tissues and 5µm sections were cut and stained with hematoxylin and eosin (HE). A pathologist without knowledge of treatment group assignment evaluated microscopic lesions. Microscopic lesion severity was scored as previously described^4,22^ and as listed in supplementary Table 2 by examining lung sections for the following: airway epithelial necrosis, attenuation, disruption; accumulation of necrotic debris and inflammatory leukocytes within the bronchiolar lumen; percentage of airways with inflammation; peribronchiolar and perivascular lymphocytic inflammation; alveolar exudate (inflammatory leukocytes/alveolar macrophages/multinucleate giant cells)/hemorrhage; thickening of alveolar septa/interstitium by inflammatory cells/edema.

### Nasal swabs

Prior to vaccination, weekly following vaccination, and each day after experimental challenge and on the day of necropsy, nasal swab samples were collected. Nasal swabs placed in minimal essential medium (MEM) with antibiotic and antimycotic (Gibco) were vortexed, samples clarified by centrifugation (800 × *g*, 15 min, 4 °C), supernatants harvested, and stored at −80 °C for virus isolation.

### Frozen lung samples

Lung tissue was collected at necropsy and stored at −80 °C. Lung homogenates were prepared by weighing tissue, making a 10% solution (w/v) in MEM, and processed using a Miltenyi gentleMACS Cell Dissociator (Miltenyi). Homogenates were transferred to a 15 ml centrifuge tube and centrifuged at 2000 rpm for 15 min at 4 °C. The clarified supernatant was harvested and 0.1 ml was used to inoculate confluent bovine turbinate cells (BT). Following a 2 h incubation at at 37 °C, 5% CO_2_, the inoculum was removed and an appropriate volume of MEM containing 10% FBS (HyClone) and an antibiotic/antimycotic solution (cMEM) was added. Samples were incubated for seven days and observed for cytopathic effect. A 10% suspension of the lung homogenate was made in MEM. The suspension was clarified by centrifugation (1000 × *g*, 30 min, 4 °C), the supernatant harvested and stored at -80 °C.

### Virus isolation

Virus isolation was performed similar to that described in previous studies^4,22^. In the present study, supernatants from nasal swabs or lung homogenates were inoculated onto confluent monolayers of BT cells and incubated for 2 h at 37 °C with 5% CO_2_. After incubation, the inoculum was aspirated and fresh supplemented medium was added. Cultures were incubated at 37 °C, 5%CO_2_ and daily observations for cytopathic effect were conducted and recorded for 7 days following inoculation of cells.

### Virus neutralization assay

Sera were serially diluted two-fold in cMEM and added to confluent BT cells in 96-well flat-bottomed plates. BRSV (600 pfu) was added to the diluted samples and incubated for 30 min at 37 °C. The tissue culture medium was removed from the cell monolayers and replaced with the diluted serum samples. The virus was allowed to infect for 90 min at 37 °C with occasional rocking, and then the cell monolayers were washed and returned to complete cell culture medium. The plates were observed for cytopathic effect daily for 7 days.

### Real-time PCR assay

Representative gross lesion and non-lesioned lung tissue from each calf were collected in RNA*later*® (Invitrogen, Life Technologies, Carlsbad, CA) and stored. Lung tissue sample RNA was isolated using the Trizol Reagent (Invitrogen, Life Technologies) according to manufacturer’s instructions. A NanoDrop 2000 spectrophotometer (Thermo Scientific, Wilmington, DE) was used to determine RNA concentration in each sample. RNA (300 ng per sample) was DNase-treated and random primers used to synthesize cDNA following manufacturer’s instructions (Invitrogen, Life Technologies). Real-time PCR was performed on a QuantStudio 5 Real-Time PCR System (Applied Biosystems, Life Technologies, ThermoFisher Scientific, Carlsbad, CA) using SYBR Green-based methodology. The amplification conditions included 95 °C for 10 min, followed by 40 cycles at 95 °C for 15 s and 60 °C for 1 min, and a final dissociation step. Each reaction tube contained 10 µl SYBR Green master mix (Applied Biosystems), 1.25 µl each of 10 µM forward and reverse primers (supplementary Table 3), 5.5 µl dH_2_0, and 2 µl of cDNA. Gene expression was determined using the 2^−ΔΔCt^ method, with reference housekeeping gene RPS9, as previously described^4^.

### Statistical analyses

GraphPad Prism version 8.4.3 (GraphPad Software, San Diego, CA) was used to perform ANOVA and post-hoc tests where appropriate. For the statistical analyses of virus isolation data, the Kruskal–Wallis test was performed. A *p* value < 0.05 was considered significant.

## Supplementary Information

Supplementary Information 1.

Supplementary Information 2.

## Data Availability

The datasets used and/or analysed during the current study are available from the corresponding author on reasonable request.
