# Supplementary material for: An intranasal recombinant NDV-BRSV Fopt vaccine is safe and reduces lesion severity in a colostrum-deprived calf model of RSV infection
Source: Sci Rep. 2022 Dec 29;12:22552. doi: 10.1038/s41598-022-26938-w (PMC9800378; doi:10.1038/s41598-022-26938-w)
Supplement: Supplementary file 1 — Supplementary Information 1. [file 41598_2022_26938_MOESM1_ESM.pdf]

An intranasal recombinant NDV-BRSV F_{opt} vaccine is safe and reduces lesion severity in a colostrum-deprived calf model of RSV infection

Randy E. Sacco[a*], Ignacio Mena[b,c], Mitchell V. Palmer[d], Russell K. Durbin[e], Adolfo García-Sastre[b,c,f,g,h], and Joan E. Durbin[e]

[a]Ruminant Diseases and Immunology Research Unit, National Animal Disease Center/USDA/ARS, 1920 Dayton Ave., Ames, IA USA
[b]Departments of Microbiology and Medicine, Icahn School of Medicine at Mount Sinai, One Gustave Levy Place, Box 1124, New York, NY
[c]Global Health and Emergent Pathogens Institute, Icahn School of Medicine at Mount Sinai, One Gustave Levy Place, Box 1124, New York, NY
[d]Infectious Bacterial Diseases Research Unit, National Animal Disease Center/USDA/ARS, 1920 Dayton Ave., Ames, IA USA 50010
[e]Department of Pathology, Rutgers-New Jersey Medical School, 185 S. Orange Ave., Newark, NJ
[f]Department of Medicine, Division of Infectious Diseases, Icahn School of Medicine at Mount Sinai, One Gustave Levy Place, Box 1124, New York, NY
[g]The Tisch Cancer Institute, Icahn School of Medicine at Mount Sinai, One Gustave Levy Place, Box 1124, New York, NY
[h]Department of Pathology, Molecular and Cell-Based Medicine, Icahn School of Medicine at Mount Sinai, One Gustave Levy Place, Box 1124, New York, NY

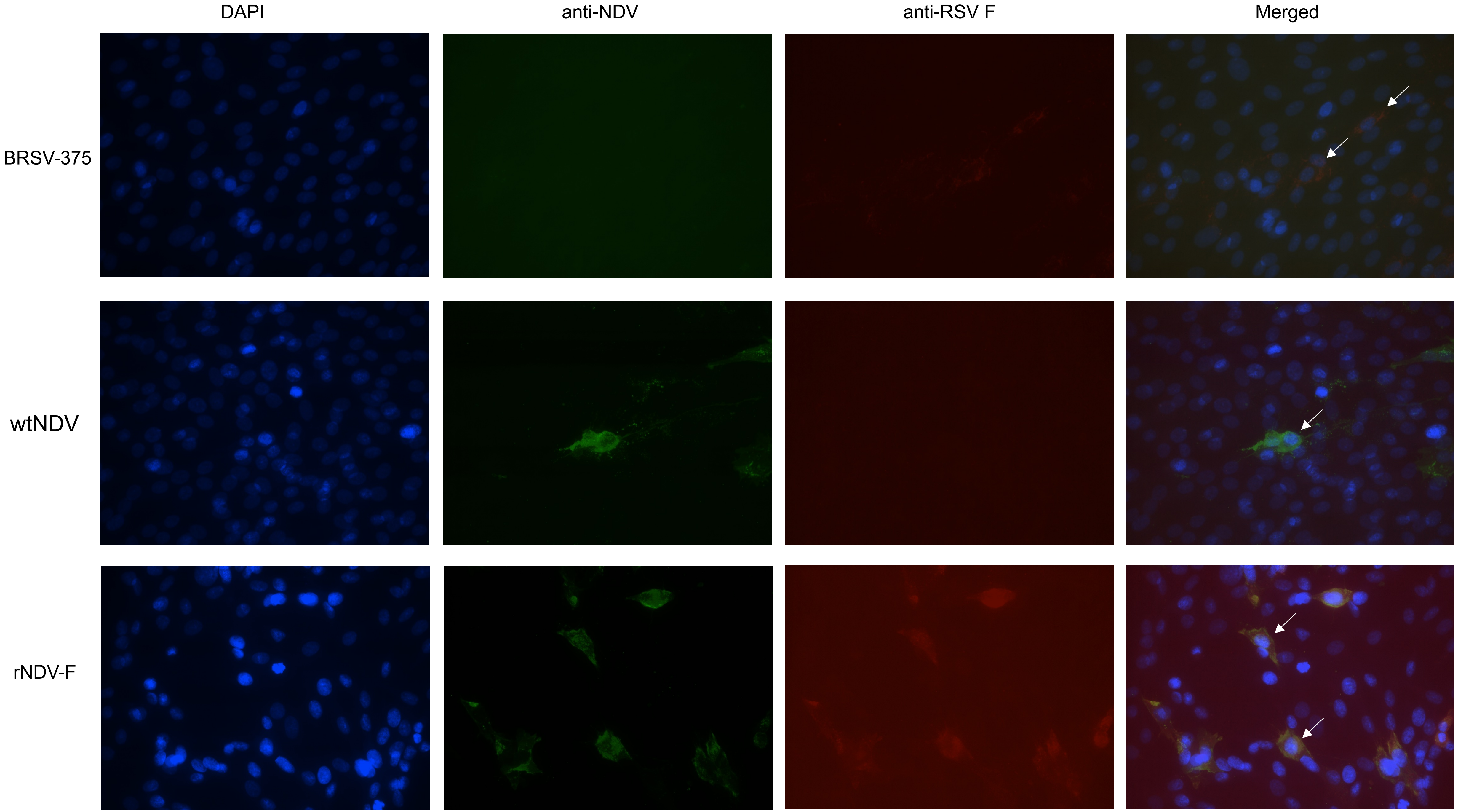
DAPI
anti-NDV
anti-RSV F
Merged
BRSV-375
wtNDV
rNDV-F

Supplementary Fig. 1. Immunofluoresence staining. Bovine turbinate (BT) cells were infected with BRSV-375, wtNDV or rNDV-BRSV F_{opt} viruses in chamber slides. Slides were washed and fixed in methanol for 10 min. Rehydrated slides with Tris buffer. Added diluted primary antibodies, washed in Tris buffer and incubated with appropriate secondary antibodies. Coverslipped with ProLong Gold Antifade Mountanat with DNA stain DAPI (blue). Anti-NDV mAb 8H2 (Serotec/Bio-Rad MCA2822;green). RSV-F mAb Palivazumab (red). Original magnification 40X.
