# Supplementary material for: An intranasal recombinant NDV-BRSV Fopt vaccine is safe and reduces lesion severity in a colostrum-deprived calf model of RSV infection
Source: Sci Rep. 2022 Dec 29;12:22552. doi: 10.1038/s41598-022-26938-w (PMC9800378; doi:10.1038/s41598-022-26938-w)
Supplement: Supplementary file 2 — Supplementary Information 2. [file 41598_2022_26938_MOESM2_ESM.docx]

**An intranasal recombinant NDV-BRSV F**_opt_ **vaccine is safe and reduces lesion severity in a colostrum-deprived calf model of RSV infection**

Randy E. Sacco^a*^, Ignacio Mena^b,c^, Mitchell V. Palmer^d^, Russell K. Durbin^e^, Adolfo García-Sastre^b,c,f,g,h^, and Joan E. Durbin^e^

***^a^Ruminant Diseases and Immunology Research Unit, National Animal Disease Center/USDA/ARS, 1920 Dayton Ave., Ames, IA USA***

***^b^Departments of Microbiology and Medicine, Icahn School of Medicine at Mount Sinai, One Gustave Levy Place, Box 1124, New York, NY***

***^c^Global Health and Emergent Pathogens Institute, Icahn School of Medicine at Mount Sinai, One Gustave Levy Place, Box 1124, New York, NY***

***^d^Infectious Bacterial Diseases Research Unit, National Animal Disease Center/USDA/ARS, 1920 Dayton Ave., Ames, IA USA 50010***

***^e^Department of Pathology, Rutgers-New Jersey Medical School, 185 S. Orange Ave., Newark, NJ***

***^f^Department of Medicine, Division of Infectious Diseases, Icahn School of Medicine at Mount Sinai, One Gustave Levy Place, Box 1124, New York, NY***

***^g^The Tisch Cancer Institute, Icahn School of Medicine at Mount Sinai, One Gustave Levy Place, Box 1124, New York, NY***

***^h^Department of Pathology, Molecular and Cell-Based Medicine, Icahn School of Medicine at Mount Sinai, One Gustave Levy Place, Box 1124, New York, NY***

| **Gross Pathology Score** | **% of lung affected** |
| --- | --- |
| 0 | Lungs free of lesions |
| 1 | 1-5% affected |
| 2 | 5-15% affected |
| 3 | 15-30% affected |
| 4 | 30-50% affected |
| 5 | >50% affected |

**Supplementary Table 1.** Scoring criteria for gross pathology in the lungs. The extent of pneumonic consolidation was evaluated using the scoring criteria outlined.

**Supplementary Table 2.** Scoring criteria for microscopic pathology in the lungs. The extent of pneumonic consolidation was evaluated using the following criteria. Each category was assigned a score from 0 (none/minimal) to 3 (severe). Scores for each category were totaled to obtain a total score out of 18. Aggregate scores are presented in Figure 2.

| **Score** | **0 (none/minimal)** | **1 (mild)** | **2 (moderate)** | **3 (severe)** |
| --- | --- | --- | --- | --- |
| **Airway epithelial necrosis/ attenuation/disruption** | NONE (Normal tall columnar ciliated epithelium) | Affecting up to 10% of airways | Affecting > 10% and <40% of airways | Affecting more than 40% of airways |
| **Accumulation of necrotic debris and inflammatory leukocytes within the bronchiolar lumen** | No/minimal inflammation | Sparsely scattered inflammatory cells affecting occasional airways | More than a few scattered intraluminal inflammatory aggregates | Bronchioles completely blocked by inflammatory/necrotic debris |
| **Percentage of airways with inflammation** | No/minimal inflammation/ Very few airways affected | Affecting up to 10% of airways | Affecting > 10% and <40% of airways | Affecting more than 40% of airways |
| **Peribronchiolar and perivascular lymphocytic inflammation** | None/minimal | Incomplete, or loosely formed cuffs of 1-2 cell layers | Numerous cuffs, predominantly incomplete and loosely-formed with lesser well formed complete cuffs of 3-4 cell layers | Numerous cuffs, predominantly  well-formed with numerous broad, dense cuffs of > 5 cell layers |
| **Alveolar exudate (inflammatory leukocytes/alveolar macrophages/multinucleate giant cells)/hemorrhage** | None/minimal | Alveoli and/or interlobular septal involvement. Affecting up to 10% of lung | Alveoli and/or interlobular septal involvement.  Affecting > 10% and <40% of lung | Alveoli and/or interlobular septal involvement. Affecting > 40% of section |
| **Thickening of alveolar septa/interstitium by inflammatory cells/edema** | Septae typically 1-2, or occasionally 3, nucleated cells wide and absence of inflammatory cells | Scattered inflammatory cells within alveolar walls Affecting < 10% of the section | Focal or multifocal alveolar septal inflammation with regions of moderate thickening of septae. Affecting > 10% and <40% | Coalescing to diffuse alveolar septal inflammation. Affecting >40% of the section |

**Supplementary Table 3**. Real-time PCR Primer Sets

|  | Forward | Reverse |
| --- | --- | --- |
| RPS9 | CGC CTC GAC CAA GAG CTG AAG | CCT CCA GAC CTC ACG TTT GTT CC |
| Muc 5B | TCT ACC TGA CCG TGG AGA CC | GTT GAT GAT GCT GCA CTG CT |
| CCL2 | CGC CTG CTG CTA TAC ATT CA | GCT CAA GGC TTT GGA GTT TG |
| CXCL8 | CGC TGG ACA GCA GAG CTC ACA AG | GCC AAG AGA GCA ACA GCC AGC T |
| CXCL9 | TCC TGA CTC TGA CTG GAG TT | GAC CTG TTT CTC CCA CTC TTT |
| CXCL10 | GTC AAG CCC TAA TTG TCC GTG GAC | AAA CTG TCA GTA GCA AGG CTG GGA |
|  |  |  |
